# Vascular endothelial growth factor +936C/T polymorphism and breast cancer risk: a meta-analysis of 13 case–control studies

**DOI:** 10.1007/s13277-013-1354-2

**Published:** 2014-01-05

**Authors:** Yulan Yan, Hongjie Liang, Taijie Li, Shihui Guo, Meng Li, Shan Li, Xue Qin

**Affiliations:** Department of Clinical Laboratory, First Affiliated Hospital of Guangxi Medical University, 6 Shuangyong Road, Nanning, 530021 Guangxi People’s Republic of China

**Keywords:** VEGF, Polymorphism, Breast cancer, Meta-analysis

## Abstract

The association between vascular endothelial growth factor (VEGF) +936C/T polymorphism and breast cancer risk has been widely reported, but results were inconsistent. In order to derive a more precise estimation of the relationship, a meta-analysis was performed. Eligible articles were identified through search of databases including PubMed, Embase, and Chinese Biomedical Literature Database (CBM). The association between the VEGF +936C/T polymorphism and breast cancer risk was conducted by odds ratios (ORs) and 95 % confidence intervals (95 % CIs). Finally, a total of 13 studies with 6,879 cases and 7,219 controls were included in our meta-analysis. Overall, a significant association was found between VEGF +936C/T polymorphisms and the risk of breast cancer in overall populations under five models (T vs. C: OR = 0.83, 95 % CI = 0.73–0.94, *P* = 0.002; TT vs. CC: OR = 0.74, 95 % CI = 0.61–0.91, *P* = 0.004, Fig. 1a; TC vs. CC: OR = 0.83, 95 % CI = 0.71–0.96, *P* = 0.014; TT vs. CC/CT: OR = 0.77, 95 % CI = 0.62–0.94, *P* = 0.010; TT/TC vs. CC: OR = 0.82, 95 % CI = 0.72–0.95, *P* = 0.006). In the subgroup analysis by ethnicity, there were also significant associations found between VEGF +936C/T polymorphism and breast cancer risk in Asians and Caucasians. In conclusion, the results of our meta-analysis suggest that the VEGF +936C/T polymorphism is significantly associated with breast cancer development and the VEGF 936T allele carriers may be associated with decreased breast cancer risk.

## Introduction

Breast cancer is the second most common cancer in women after skin cancer, accounting for about one third of all cancers in women [1]. Global breast cancer incidence has been increasing by more than one million new cases every year, and the incidence is significantly higher in developed countries than in developing countries [2]. Incidence of breast cancer is increasing around the world, accounting for 23 % of the total cancer cases and 14 % of the cancer deaths in 2008. It is still the leading cause of cancer mortality in women [3]. Although the mechanism of breast carcinogenesis is still not fully understood, it is well known that tumorigenesis is a multistep process with multifactors involved [4, 5]. Besides environmental factors [6, 7], gene-based factors leading to individuals’ susceptibility to cancer development have been commonly studied [8, 9]. Gene polymorphisms, common risk factors for breast cancer, have been extensively studied recently [10, 11].

Previous studies have suggested that low-penetrance susceptibility genes combined with environmental factors may be important in the development of cancers, including breast cancer [7], while vascular endothelial growth factor (VEGF), which is located at 6p21.3, containing eight exons and seven introns, is one of the low-penetrance susceptibility genes [12]. At least 30 single-nucleotide polymorphisms (SNP) in this gene have been described in the publications. Among these, one of the most common gene, VEGF +936C/T (rs3025039) in the 3′-untranslated region, was found to be associated with variations in VEGF protein production [13, 14]. It was reported that the C-to-T change led to the loss of a potential binding site for transcription factor AP-4 and that VEGF plasma levels in 936T allele carriers were significantly lower than those in non-carriers; besides, the T allele has been found to be associated with a reduced uptake of ^18^F-fluorodeoxyglucose, used for detection and staging of breast cancer [15–18].

Up to now, a number of studies have reported the association between the VEGF +936C/T polymorphism and breast cancer susceptibility, but the results remain inconsistent [19–29]. In order to estimate the association between VEGF +936C/T polymorphism and breast cancer susceptibility, we conducted this meta-analysis.

## Methods

### Search strategy

We performed an electronic search of the PubMed, Embase, and Chinese Biomedical Literature Database (CBM) to retrieve articles linking VEGF +936C/T gene polymorphism and susceptibility to breast cancer available until August 2013 with keywords “breast cancer,” “breast neoplasm,” “vascular endothelial growth factor,” “VEGF,” “polymorphism,” and “variant,” and there were no limitations to the language of publications. Additional studies were identified by a hand search of the references of original studies, and review articles were also examined to find additional eligible studies.

### Inclusion and exclusion criteria

Eligible studies included in our meta-analysis had to meet the following criteria: (a) they should be case–control studies, (b) they should evaluate the VEGF +936C/T polymorphism and breast cancer risk, (c) they should supply the available genotype frequency in cases and controls, and (d) they should have sufficient published data for estimating an odds ratio (OR) with 95 % confidence interval (CI). The exclusion criteria were as follows: (a) not a case–control study, (b) no usable data reported, (c) duplicate data, (d) abstract, comment, review, and editorial. When multiple publications reported on the same or overlapping data, the most recent or largest population was selected.

### Data extraction

Two investigators independently extracted the data, based on the inclusion criteria mentioned above. If conflicting evaluations were encountered, an agreement was reached following a discussion; if agreement could not be reached, then a third author was consulted to resolve the debate. The following information were extracted: (a) the name of the first author, (b) year of publication, (c) country of origin, (d) ethnicity, (e) genotyping methods, (f) source of the control group, (g) distribution of genotypes in case and control groups. We also evaluated whether the genotype distributions were in the Hardy–Weinberg equilibrium.

### Statistical analysis

The odds ratio (OR) and 95 % confidence interval (CI) were used to evaluate the strength of associations between the VEGF +936C/T polymorphism and the risk of breast cancer according to five genetic models: allele contrast (T vs. C), homozygote (TT vs. CC), heterozygote (TC vs. CC), recessive (TT vs. TC/CC), and dominant (TT/TC vs. CC) models. The heterogeneity was tested by a chi-square-based *Q* statistic test. The effect of heterogeneity was quantified by using *I* ^2^ values as well as *P* values [30]. If *I* ^2^ value <50 % and *P* > 0.10, this suggests that obvious heterogeneity does not exist, ORs were pooled by a fixed-effects model (the Mantel–Haenszel method) [31]. Otherwise, ORs were pooled by a random-effects model (DerSimonian and Laird method) [32].

The Hardy–Weinberg equilibrium (HWE) [33] of controls was tested by using a professional web-based program (http://ihg2.helmholtz-muenchen.de/cgibin/hw/hwa1.pl); if *P* > 0.05, this suggests that the controls followed the HWE balance. Sensitivity analysis was performed to evaluate the stability of the results. A single study involved in the meta-analysis was omitted each time to reflect the influence of the individual data set on the pooled ORs [34]. When the Hardy–Weinberg equilibrium disequilibrium existed (*P* < 0.05 was considered statistically significant), the sensitivity analysis was also conducted. Possible publication bias was tested by Egger’s test (*P* < 0.05 was considered representative of statistically significant publication bias) [35] and visual observation of funnel plot [36] in the meta-analysis. All statistical tests were performed with STATA Software (version 9.2, Stata Corp). *P* < 0.05 for any test or model was considered to be statistically significant.

## Results

### Search results and study characteristics

After careful examination according to the inclusion criteria, a total of 11 publications with 13 studies including 6,879 cases and 7,219 controls were included in our meta-analysis [19–29]. In the 13 studies, controls were mainly healthy populations and matched for age, and the genotype distribution in the controls of all studies was consistent with HWE (all *P* > 0.05). The main characteristics of the studies included in the present meta-analysis are listed in Table 1.

**Table 1:** Characteristics of case–control studies included in VEGF +C936T polymorphism and breast cancer risk

First author	Year	Country	Ethnicity	Genotyping methods	Source of control	Cases			Controls		
						CC	CT	TT	CC	CT	TT
Luo	2013	China	Asian	PCR-RFLP	HB	446	210	24	426	204	50
Rodrigues	2012	Span	Caucasian	PCR-RFLP	PB	366	76	5	332	99	11
Absenger	2013	Austria	Caucasian	TaqMan	PB	371	138	12	580	201	20
Oliveira	2011	Brazil	Mix	PCR-RFLP	PB	190	43	2	176	52	7
Lin	2009	China	Asian	PCR-RFLP	HB	155	59	6	211	117	6
Jakubowska	2008	Poland	Caucasian	PCR-RFLP	PB	245	67	7	202	81	7
Balasubramanian	2007	England	Caucasian	TaqMan	PB	624	204	20	531	165	12
Kataoka	2006	China	Asian	Taqman	PB	744	334	31	793	351	51
Jacobs	2006	USA	Mix	Taqman	PB	360	110	10	353	111	15
Jin(a)	2005	Polish	Caucasian	PCR-RFLP	PB	298	10	14	297	114	11
Jin(b)	2005	German	Caucasian	PCR-RFLP	PB	120	31	2	128	31	4
Jin(c)	2005	Swenden	Caucasian	PCR-RFLP	PB	708	204	12	720	203	11
Krippl	2003	Austria	Caucasian	Taqman	PB	412	79	9	353	137	10

*PCR-RFLP* polymerase chain reaction–restriction fragment length polymorphism, *HB* hospital based, *PB* population based

### Meta-analysis results

**Fig. 1:**
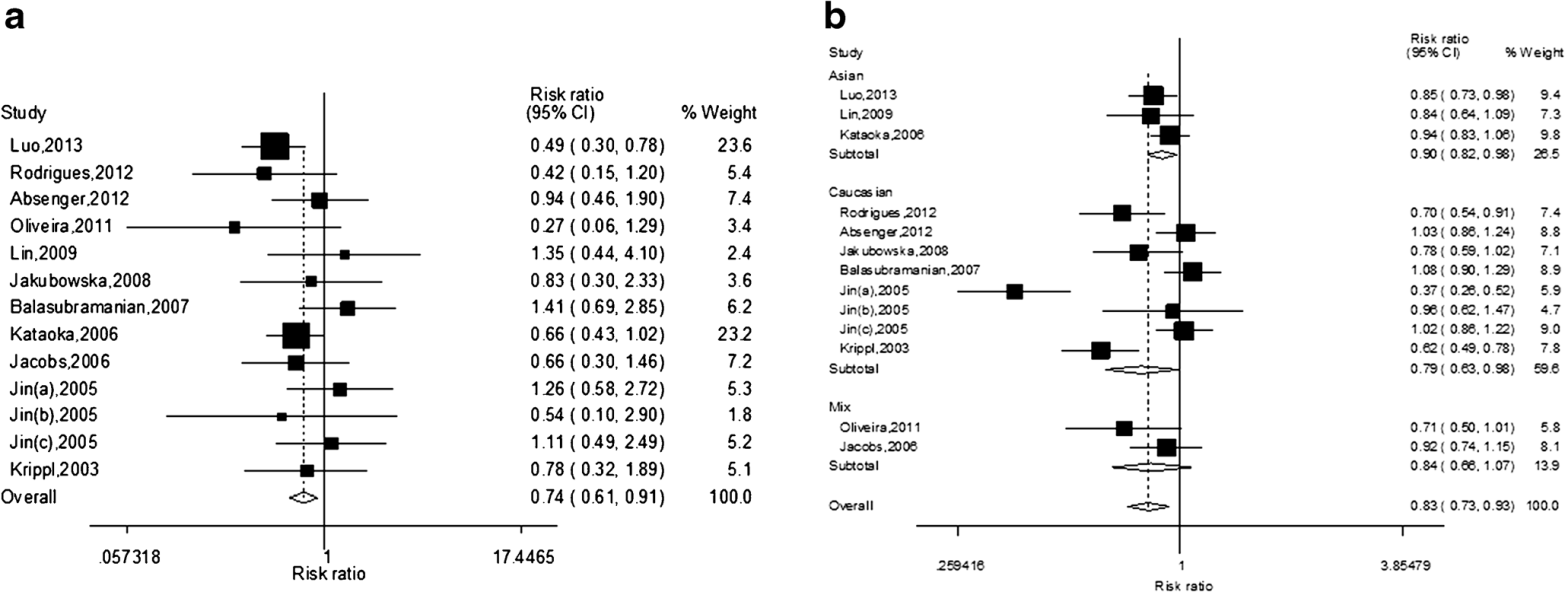
**a** The forest plot describing the meta-analysis under the homozygous model for the association between VEGF +936C/T polymorphism and the risk of breast cancer in overall population (TT vs. CC). **b** The forest plot describing the meta-analysis under the allele model for the association between VEGF +936C/T polymorphism and the risk of breast cancer in the subgroup analysis based on ethnicity (T vs. C)

The main results of this meta-analysis and the heterogeneity test were shown in Table 2. A significant association was found between VEGF +936C/T polymorphism and the risk of breast cancer in overall populations under five models (T vs. C: OR = 0.83, 95 % CI = 0.73–0.94, *P* = 0.002; TT vs. CC: OR = 0.74, 95 % CI = 0.61–0.91, *P* = 0.004, Fig. 1a; TC vs. CC: OR = 0.83, 95 % CI = 0.71–0.96, *P* = 0.014; TT vs. CC/CT: OR = 0.77, 95 % CI = 0.62–0.94, *P* = 0.010; TT/TC vs. CC: OR = 0.82, 95 % CI = 0.72–0.95, *P* = 0.006). In the subgroup analysis by ethnicity, there were also significant associations found between VEGF +936C/T polymorphisms and breast cancer risk in Asians (T vs. C: OR = 0.90, 95 % CI = 0.82–0.98, *P* = 0.014, Fig. 1b; TT vs. CC: OR = 0.61, 95 % CI = 0.45–0.83, *P* = 0.002; TT vs. CC/CT: OR = 0.61, 95 % CI = 0.45–0.83, *P* = 0.002) and Caucasians (T vs. C: OR = 0.79, 95 % CI = 0.64–0.99, *P* = 0.036, Fig. 1b; TC vs. CC: OR = 0.74, 95 % CI = 0.56–0.97, *P* = 0.027; TT/TC vs. CC: OR = 0.77, 95 % CI = 0.60–0.98, *P* = 0.031).

**Table 2:** Results of meta-analysis for VEGF +C936T polymorphism and breast cancer risk

Comparison	Population	Number	Test of association			Model	Test of heterogeneity	
			OR	95 % CI	*P*		*P*	*I* ^2^
T vs. C	Overall	13	0.83	0.73–0.94	0.002	R	0	76.7
	Asian	3	0.90	0.82–0.98	0.014	F	0.488	0
	Caucasians	8	0.79	0.64–0.99	0.036	R	0	85.5
	Mix	2	0.85	0.71–1.03	0.097	F	0.226	31.7
TT vs. CC	Overall	13	0.74	0.61–0.91	0.004	F	0.316	12.8
	Asian	3	0.61	0.45–0.83	0.002	F	0.225	32.9
	Caucasians	8	0.96	0.71–1.29	0.767	F	0.679	0
	Mix	2	0.54	0.27–1.07	0.078	F	0.316	0.5
TC vs. CC	Overall	13	0.83	0.71–0.96	0.014	R	0	83.3
	Asian	3	0.97	0.88–1.06	0.498	F	0.186	40.5
	Caucasians	8	0.74	0.56–0.97	0.027	R	0	89.4
	Mix	2	0.92	0.76–1.12	0.426	F	0.384	0
TT vs. TC/CC	Overall	13	0.77	0.62–0.94	0.010	F	0.177	26.5
	Asian	3	0.61	0.45–0.83	0.002	F	0.163	44.9
	Caucasians	8	1.02	0.76–1.38	0.886	F	0.607	0
	Mix	2	0.55	0.27–1.09	0.087	F	0.342	0
TT/TC vs. CC	Overall	13	0.82	0.72–0.95	0.006	R	0	81.8
	Asian	3	0.94	0.86–1.02	0.121	F	0.350	4.6
	Caucasians	8	0.77	0.60–0.98	0.031	R	0	88.6
	Mix	2	0.89	0.74–1.07	0.213	F	0.287	11.7

*OR* odds ratio, *CI* confidence interval, *F* fixed-effects model, *R* random-effects model

### Sensitivity analysis

Sensitivity analyses were conducted to determine whether modification of the inclusion criteria of the meta-analysis affected the final results. A single study involved in the meta-analysis was deleted each time to reflect the influence of the individual data set on the pooled OR, and the corresponding pooled OR was not materially altered (data not shown), indicating that our results were relatively stable and credible.

### Publication bias

**Fig. 2:**
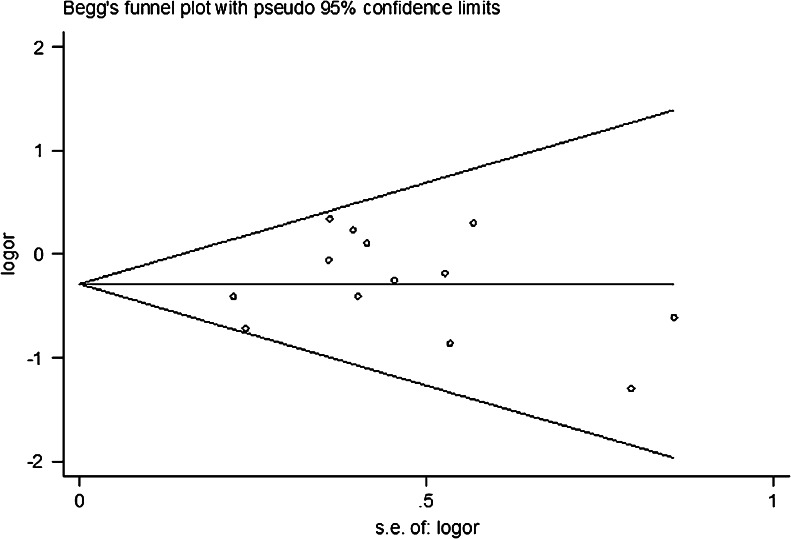
Begg funnel plot for publication bias test for the association between VEGF +936C/T polymorphism and the risk of breast cancer under the homozygous model (TT vs. CC). Each point represents a separate study for the indicated association. Log [OR], natural logarithm of OR. Horizontal line means effect size

A funnel plot and Egger’s test were performed to assess publication bias. The funnel plot is relatively straightforward in observing whether the publication bias is present, and Egger’s test was used to provide statistical evidence of symmetries of the plots. The shape of the funnel plot did not reveal any evidence of obvious asymmetry (Fig. 2). Similarly, the results of Egger’s test still did not suggest any evidence of publication bias (all *P* > 0.05, data not shown).

## Discussion

Angiogenesis is essential for the growth of microscopic cancers into larger tumors, while VEGF plays a central role in angiogenesis through a variety of mechanisms such as effects on endothelial cell proliferation, survival, and migration [37, 38]. Previous studies suggested that VEGF 936T allele causes lower VEGF plasma levels, mainly by the following two mechanisms. One is that the 936C/T mutation results in the loss of a potential binding site for AP-4. AP-4 is a helix–loop–helix transcription factor that enhances the expression of several viral and cellular genes by binding to specific enhancer sites. The other one is that the association between the 936C/T mutation and VEGF plasma levels could be due to linkage disequilibrium between this mutation and another yet unknown functional mutation elsewhere in the VEGF gene sequence [16, 39–41]. Thus, VEGF 936T allele carriers were considered to be associated with decreased breast cancer risk.

The present meta-analysis, including 6,879 cases and 7,219 controls, explored the association between the VEGF +936C/T polymorphism and breast cancer risk. The results show that the VEGF +936C/T polymorphism is significantly associated with breast cancer development. Actually, it might act as a protective factor for breast cancer. In the subgroup analyses based on ethnicity, a significant association was found in Asian and Caucasian populations. This may be explained by the fact that cancer is a complicated multigenetic disease and different genetic backgrounds may contribute to the discrepancy.

Although a comprehensive analysis was conducted to show the association between MTHFR polymorphism and risk of breast cancer, there are still some limitations that should be pointed out. First, the number of studies and the number of samples included in the meta-analysis were relatively small. Second, in the subgroup analyses, the number of Asians was relatively small, not having enough statistical power to explore the real association. Additionally, no data were available about Africans. Third, the controls were not uniformly defined. Some studies used controls that were population based, while others used hospital-based controls, which may not be representative of the general population. Finally, our results were based on unadjusted estimates, while a more precise analysis should be conducted if individual data were available, which would allow for the adjustment of other co-variants including age, menopausal status, environmental factors, and lifestyle.

In conclusion, the results of our meta-analysis suggest that the VEGF +936C/T polymorphism is significantly associated with breast cancer development and the VEGF 936T allele carriers may be associated with decreased breast cancer risk.
